# Integrative Genomic Analysis Identifies That *SERPINA6*-rs1998056 Regulated by FOXA/ERα Is Associated with Female Hepatocellular Carcinoma

**DOI:** 10.1371/journal.pone.0107246

**Published:** 2014-09-08

**Authors:** Na Shen, Jing Gong, Ying Wang, Jing Tian, Jiaming Qian, Li Zou, Wei Chen, Beibei Zhu, Xinghua Lu, Rong Zhong, Anyuan Guo, Li Wang, Xiaoping Miao

**Affiliations:** 1 State Key Laboratory of Environment Health (Incubation), MOE (Ministry of Education) Key Laboratory of Environment & Health, Ministry of Environmental Protection Key Laboratory of Environment and Health (Wuhan), and Department of Epidemiology and Biostatistics, School of Public Health, Tongji Medical College, Huazhong University of Science and Technology, Wuhan, China; 2 Department of Epidemiology and Biostatistics, Institute of Basic Medical Sciences, Chinese Academy of Medical Sciences; School of Basic Medicine, Peking Union Medical College, Beijing, China; 3 Wuhan Centers for Disease Prevention and Control, Wuhan, China; 4 Division of Gastroenterology, Peking Union Medical College Hospital, Chinese Academy of Medical Sciences, Peking Union Medical College, Beijing, China; 5 Hubei Bioinformatics and Molecular Imaging Key Laboratory, Department of Systems Biology, College of Life Science and Technology, Huazhong University of Science and Technology, Wuhan, China; Beijing University of Chemical Technology, China

## Abstract

The human forkhead box A1 (FOXA1) and A2 (FOXA2) transcription factors have been found to control estrogen and androgen signaling through co-regulating target genes with sex hormone receptors. Here we used an integrative strategy to examine the hypothesis that genetic variants at FOXA1/2 binding elements may be associated with sexual dimorphism of hepatocellular carcinoma (HCC) risk. Firstly we extracted chromatin immunoprecipitation-sequencing (ChIP-seq) data of FOXA1, FOXA2 and estrogen receptor 1(ERα) from ENCODE database to obtain dual target regions of FOXA/ERα, and further intersected these regions with genes’ promoters. Then we used MATCH program to predict FOXA binding elements, in which genetic variants were retrieved by dbSNP database (NCBI, build 134). A total of 15 candidate variants were identified in this stage. Secondly we performed a case-control study with 1,081 HCC patients and 2,008 matched controls and found a significant association of *SERPINA6*-rs1998056 with female HCC risk under common genetic models (e.g. GG versus CC: OR = 2.03, 95% CI = 1.26–3.27, *P* = 0.004). Moreover, results from our real-time quantitative polymerase chain reaction (qPCR) using 72 normal liver tissues adjacent to the tumors showed that *SERPINA6* expression was significantly different among different genotypes of this variant (GG versus CC: *P* = 0.032; Group test: *P* = 0.060). In summary, our study suggested that *SERPINA6*-rs1998056 regulated by FOXA/ERα might be associated with female HCC risk.

## Introduction

Hepatocellular carcinoma (HCC) is one of the most frequently diagnosed malignancies. In the worldwide, it is the fifth common cancer in men and the seventh in women, with an estimated 748,300 new cases and 695,900 deaths in 2008 [1]. More than half of these suffers were considered to occur in China [2]. Previous epidemiology studies have revealed several environmental hazards, such as chronic hepatitis B or C virus infection, aflatoxin B1 intake, obesity and alcoholism [2]–[6]. But only a few exposed individuals actually develop HCC during their lifetimes, suggesting that genetics also play an important role in the pathogenesis of this disease. Interestingly, HCC demonstrates the significant difference of incidence between men and women [1]. Although different lifestyles and environmental exposures can explain a part of sexual dimorphism of HCC, biological inequality between men and women also derives from subtle regulations of genetic factors and demonstrates a variety of hormone-related conditions on health and disease.

The human forkhead box A 1 (*FOXA1*) and forkhead box A2 (*FOXA2*) are two members of the *FOX* gene family that encode a set of transcription factors playing important roles in controlling cell growth, proliferation and differentiation [7]. Accumulative studies have indicated that *FOXA1* and *FOXA2*, as well as their orthologous members *Foxa1* and *Foxa2* in mice, are associated with several tumors, especially with the hormone-dependent cancers [8]–[13]. It is generally considered that the recruitment of estrogen receptor 1 (ERα) or androgen receptor (AR) to their target genes depend on the modulation of FOXA1 in breast or prostate cancer, respectively, and FOXA factors may be essential in estrogen and androgen signaling in hormone-dependent malignancies [14]–[17]. HCC is a sexually dimorphic disease related to sex hormone too. Recently, Li Z et al. identified the similar and central role of Foxa1 and Foxa2 in regulating estrogen and androgen signaling through recruitment of ERα and AR to their targets in the carcinogenesis of liver, thereby providing an explicit explanation for sexual dimorphism of HCC [18]. Nevertheless, the association between genetic variants at FOXA binding elements and the development of HCC was implied by their preliminary results in several human samples [18]. To further investigate the genetics of sexual dimorphism contributing to HCC risk, large-scale association studies are warranted.

Informatively, we used an integrative genomic strategy to examine the hypothesis that genetic variants at FOXA1/2 binding elements likely relate to sexual dimorphism of HCC risk. In the stage of bioinformatic data mining, we extracted chromatin immunoprecipitation-sequencing (ChIP-seq) data of FOXA1, FOXA2 and ERα from online databases to obtain dual target regions of FOXA/ERα, and further intersected these regions with genes’ promoters. Then we used MATCH program to predict FOXA binding elements, in which candidate variants were retrieved by dbSNP database (NCBI, build 134). In the stage of case-control study, 1,081 patients with newly diagnosed HCC and 2,008 matched tumor-free controls were enrolled to examine the effects of selected candidate variants on HCC risk. Finally in the stage of functional experiment, a real-time quantitative polymerase chain reaction (qPCR) was carried out to evaluate the effect of rs1998056 on *SERPINA6* expression using 72 normal liver tissues adjacent to the tumors with different genotypes.

## Materials and Methods

### Identification of candidate variants

Two import results from Li Z et al. [18] were applied to our study: (1) Foxa1 and Foxa2 conduct the redundant regulation in liver and there are no significant differences of either Foxa1 or Foxa2 binding between males and females. That was the theoretical basis for us to combine ChIP-seq data of FOXA1 and FOXA2 from HepG2 cell line. (2) The vast majority of the dual targets (76% of Foxa/ERα and 64% of Foxa/AR) are overlapped between males and females. So we not only examined candidate variants in total population, but also detected their effects in both men and women.

**Figure 1:**
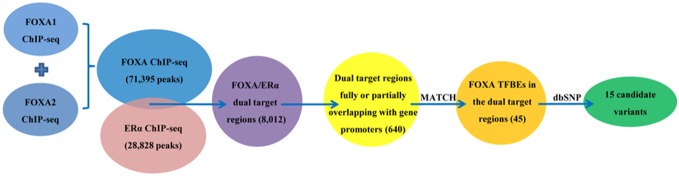
A computational strategy for identifying candidate variants at FOXA1/2 binding elements.

The schematic overview of identifying candidate variants at FOXA1/2 binding elements is shown in Figure 1. First, ChIP-seq data of FOXA1, FOXA2 and ERα were downloaded from the Encyclopedia of DNA Elements (ENCODE) Project (http://genome.ucsc.edu/ENCODE/downloads.html) to extract corresponding sites of these transcription factors (TFBSs). Specifically, genome-wide TFBSs of FOXA1and FOXA2 were extracted from HepG2 cell line; genome-wide TFBSs of ERα were extracted from ECC-1 and T47-D cell lines due to lacking of ERα ChIP-seq data of HepG2 cell line. For the replicated experiments in the same cell line, overlapping peak locations were selected; for the independent experiments in different cell lines, peak locations were put together for further analysis. In consideration of the redundant regulation of FOXA1 and FOXA2 in liver [18], we combined the ChIP-Seq results of FOXA1 and FOXA2 together. Thus we got two TFBS datasets of FOXA (71,395 peaks) and ERα (28,828 peaks). Second, dual target regions of FOXA/ERα were identified as follows. Each TFBS genome location of FOXA was compared with that of ERα. If the distance between FOXA TFBS and ERα TFBS was less than 250 bp, we defined the corresponding regions as dual target regions. In this step, we obtained a total of 8,012 dual target regions of FOXA/ERα. Because the TFBSs within or near gene’s promoter regions are generally considered more likely to be real binding sites, the third step was to intersect dual target regions of FOXA/ERα with gene’s promoter regions to find the potentially functional dual target regions. We downloaded all genes’ genomic locations from the University of California Santa Cruz (UCSC) Genome Browser (http://genome.ucsc.edu/) and then defined their upstream 5 kb regions as genes’ promoter regions. By mapping the locations of these TFBSs to genes’ promoters, a total of 640 potentially functional dual target regions of FOXA/ERα were identified. Specially, these 640 dual target regions fully or partially overlapped with promoter regions. To further narrow down the dual target regions, we further fine mapped the TFBSs of FOXA and ERα in their dual target regions by MATCH program, which can finely predict TFBSs based on the positional weight matrices from the TRANSFAC database [19]. The TFBSs predicted by MATCH program were defined as transcriptional factor binding elements (TFBEs) in our study. Thus we got 45 FOXA TFBEs in the dual target regions overlapping genes’ promoters. Finally, candidate variants were searched in these regions by retrieving human reference SNP database (downloaded from dbSNP database of NCBI, build 134). Ultimately, we identified a total of 15 genetic variants, which are co-regulated by FOXA/ERα, located at FOXA TFBEs near or within genes’ promoter regions (Table S1).

All bioinformatic processes above were conducted by in-house *Perl* scripts. Of 15 candidate variants, there were 3 common variants with minor allele frequencies (MAFs) more than 0.05. They were rs111570813 (in TFBE of gene inter-alpha-trypsin inhibitor heavy chain 2, *ITIH2*), rs1998056 (in TFBE of gene serpin peptidase inhibitor, clade A (alpha-1 antiproteinase, antitrypsin), member 6, *SERPINA6*) and rs35335725 (in TFBE of gene keratin associated protein 2–3, *KRTAP2-3*). Because research about *KRTAP2-3* is rare, we selected *ITIH2*-rs111570813 and *SERPINA6*-rs1998056 for population association analysis. However, the custom probe for rs111570813 is failed due to the sequence around this locus with high GC content. Thus, only *SERPINA6*-rs1998056 of TaqMan SNP Genotyping Assay was successfully synthesized for the following validation.

### Study subjects

This study consisted of 1,081 incident patients with HCC and 2,008 cancer-free controls. All subjects were unrelated ethnic Han Chinese. HCC patients were newly diagnosed and histologically confirmed, without treatment of chemotherapy or radiotherapy prior to surgery. Among 1,081 patients included, 1,009 cases providing whole blood samples were consecutively recruited between January 2010 and March 2013 at Tongji Hospital of Huazhong University of Science and Technology (HUST), Wuhan, Central China, and 72 cases providing normal liver tissues adjacent to the tumors were enrolled from Peking Union Medical College Hospital (PUMCH), Beijing, China. These liver tissues were obtained from surgically removed specimens of individual patients. They were immediately placed in liquid nitrogen after resection, and then transferred to a −80°C freezer for storage until analysis. Controls providing whole blood samples were randomly selected from the healthy examination population in the Wuhan area during the same time period as the cases were enrolled, part of which also included in our previous case-control studies [20]–[22]. The inclusion criteria for controls contained no individual history of cancer, and frequency matched to cases on the basis of age (±5 years) and gender. At recruitment, written informed consent was obtained from each study subject, and demographic data including gender, age, smoking and drinking habits were collected. This study was conducted under the approval of the institutional review boards of Tongji Medical College of HUST.

### Genotyping assay

Genomic DNA (gDNA) from 5 ml anticoagulant peripheral blood was extracted with the RelaxGene Blood DNA System DP319-02 (TIANGEN, Beijing, China). For each sample of frozen normal liver tissues adjacent to the tumors, we first pulverized it manually in liquid nitrogen and then isolated DNA using the standard phenol–chloroform method.

Candidate variant rs1998056 was genotyped using TaqMan Polymerase chain reaction (PCR) Assay (Applied Biosystems, Foster city, CA). The PCR program was heating for 10 minutes at 95°C; followed by 50 cycles of 15 seconds at 92°C and 90 seconds at 60°C. After PCR amplification, the ABI Prism 7900HT Sequence Detection System (Applied Biosystems, Foster city, CA) was applied to read the reacted plates and analyze the endpoint fluorescence to make allelic discrimination. Quality control was monitored by including 5% duplicates and negative controls, with the 100% concurrence rate of the duplicate sets. The genotyping call rate for rs1998056 was 99.4%.

### Analysis of SERPINA6 mRNA levels

Total RNA was extracted with TRIzol reagent (Invitrogen Corp., Carlsbad, CA) and then complementary DNA (cDNA) was prepared using PrimeScript 1st Strand cDNA Synthesis Kit (Takara Biotechnology (Dalian) Co., Ltd.). qPCR was carried out to quantify the relative gene expression of *SERPINA6*, using ABI Prism 7900HT Sequence Detection System (Applied Biosystems, Foster city, CA) based on the SYBR-green method. All quantifications were performed using *ACTB* as an internal reference gene. The primers used for *SERPINA6* were 5′-CTTCTATGTGGACGAGACAACTG-3′ (forward primer) and 5′-CCCACGTAGTTCATCTGCAC-3′ (reverse primer); and for *ACTB* were 5′-CATGTACGTTGCTATCCAGGC-3′ (forward primer) and 5′-CTCCTTAATGTCACGCACGAT-3′ (reverse primer). All these sequences of primers were downloaded from PrimerBank (http://pga.mgh.harvard.edu/primerbank/index.html). In a volume of 10 ul qPCR reaction, 1 ul cDNA template was mixed with 5 ul 2× Power SYBR Green PCR Master Mix (Applied Biosystems, Foster city, CA), 200 nM of paired primers and distilled water. qPCR amplification included enzyme activation at 95°C for 10 minutes, and 40 cycles of denaturing at 95°C for 15 seconds and annealing at 60°C for 1 minute. Then a dissociation stage was used to analyze the melting curves. Each sample for a given gene was analyzed in duplicate and any sample with coefficient of variance >5% were re-analyzed.

### Statistical analysis

Hardy-Weinberg Equilibrium (HWE) for genotype of rs1998056 was evaluated by using a goodness-of-fit χ^2^ test in the control group (*P* = 0.965). Either the independent-samples *t* test or the Pearson’s χ^2^ test was applied to assess demographic differences between cases and controls in the distribution of age, gender, smoking and drinking status. The odds ratio (OR) and 95% confidence interval (95% CI) were estimated by logistic regression (LR) model to investigate the association between *SERPINA6*-rs1998056 and HCC risk. Because we aimed to examine the potential effects of candidate variant in the sexual dimorphism of HCC risk, both overall analyses and subgroup analyses by gender were conducted by univariate and multivariate LR analyses. Besides, relative gene expression of *SERPINA6* was calculated by 2^−ΔCt^ method [23] where ΔC_t_ = C_t *SERPINA6*_–C_t *ACTB*_. Kruskal-Wallis one-way ANOVA test or Mann-Whitney test was performed to examine the difference between *SERPINA6* expression levels and rs1998056 genotype carriers. All the statistical analyses were conducted by PASW Statistics 18.0 software (IBM Corporation, Somers, New York) and all *P* values were two-sided with the statistical significance criteria of *P*<0.05. In addition, we computed the statistic power using the Power V3.0 (http://dceg.cancer.gov/tools/design/POWER) and got a power of 0.94 to detect an OR of 1.30 in our sample size.

## Results

### Variants selection and subject characteristics

By using a computational strategy shown in Figure 1, we identified a total of 15 genetic variants at FOXA1/2 TFBEs. These candidate variants are co-regulated by FOXA/ERα, and near or within genes’ promoter regions. They are involved in 8 genes (*LINC00862*, *HES1*, *ITIH2*, *BATF*, *DTD2*, *SERPINA6*, *LOC100507217* and *KRTAP2-3*), and details were presented in Table S1. There are 3 common variants with MAF>0.05, *ITIH2*-rs111570813, *SERPINA6*-rs1998056 and *KRTAP2-3*-rs35335725. Because of rare relevant report for *KRTAP2-3* and probe designed failure for *ITIH2*-rs111570813, only *SERPINA6*-rs1998056 was further analyzed in the following study.

In the subsequent case-control study, demographic characteristics of 1,081 HCC patients and 2,008 cancer-free controls are presented in Table 1. There was no significant difference between patients and controls in the distribution of age (*P* = 0.149) and gender (*P* = 0.937). However, significantly more smokers were presented among cases than among controls (59.9% versus 48.9%, *P* = 1.611×10^−9^). Similar result was also observed in drinking status (52.9% versus 44.7%, *P* = 2.583×10^−6^).

**Table 1:** Distributions of characteristics among HCC patients and controls in our study.

	Cases (n = 1,081)	Controls (n = 2,008)	*P* value
	N (%)	N (%)	
Age (Mean ± SD)	54.96±11.37	55.56±10.28	0.149
Gender			0.937
Male	866 (80.1)	1,611 (80.2)	
Female	215 (19.9)	397 (19.8)	
Smoking status			1.611×10^−9^
Never	419 (38.8)	1,011 (50.3)	
Ever	647 (59.9)	982 (48.9)	
Unknown	15 (1.4)	15 (0.7)	
Drinking status			2.583×10^−6^
Never	486 (45.0)	1,092 (54.4)	
Ever	572 (52.9)	898 (44.7)	
Unknown	23 (2.1)	18 (0.9)	

Abbreviations: SD, standard deviation.

### Association between *SERPINA6*-rs1998056 and HCC risk

A total of 3,089 unrelated ethnic Han Chinese people were genotyped to examine the association between *SERPINA6*-rs1998056 and HCC risk (1,081 cases and 2,008 controls). Results of overall and subgroup analyses are summarized in Table 2. There was no statistical relation of this variant with HCC risk in total participants in genotype, recessive, dominant and additive models by using LR analyses. Similar results were also observed in males. But in females, both univariate and multivariate analyses strongly suggested a significant association between *SERPINA6*-rs1998056 and the susceptibility to HCC in all genetic models. After adjusting for age, smoking and drinking status, women with GG genotype suffered 2-fold HCC risk than those with CC genotype (OR = 2.03, 95% CI = 1.26–3.27, *P* = 0.004). Other genetic models exhibited consistent results too (additive model: OR = 1.42, 95% CI = 1.12–1.81, *P* = 0.004; recessive model: OR = 1.64, 95% CI = 1.10–2.43, *P* = 0.015; dominant model: OR = 1.60, 95% CI = 1.08–2.36, *P* = 0.019).

**Table 2:** Association analyses between *SERPINA6*-rs1998056 and HCC risk in different genetic models.

Geneticmodels	Overall				Male				Female			
	Cases/Controls	Crude OR(95%CI)	Adjusted OR(95%CI)*	*P* *	Cases/Controls	Crude OR(95%CI)	Adjusted OR(95%CI)†	*P* †	Cases/Controls	Crude OR(95%CI)	Adjusted OR(95%CI)†	*P* †
Genotype	1,072/1,997			0.765	857/1,601			0.504	215/396			**0.015**
CC	280/538	Reference	Reference		229/405	Reference	Reference		51/133	Reference	Reference	
CG	551/998	1.06(0.89, 1.27)	1.07(0.89, 1.29)	0.679	448/815	0.97(0.78, 1.19)	1.00(0.81, 1.23)	0.994	103/183	1.47 (0.98, 2.20)	1.41 (0.93, 2.14)	0.109
GG	241/461	1.00(0.81, 1.24)	1.05(0.84, 1.30)	0.917	180/381	0.84(0.66, 1.06)	0.89(0.69, 1.13)	0.329	61/80	**1.99** **(1.25, 3.16)**	**2.03** **(1.26, 3.27)**	**0.004**
Additive	1,072/1,997			0.655	857/1,601			0.344	215/396			**0.004**
CC	280/538	Reference	Reference		229/405	Reference	Reference		51/133	Reference	Reference	
CG	551/998	1.00(0.90, 1.12)	1.03(0.92, 1.14)		448/815	0.92(0.81, 1.03)	0.94(0.83, 1.07)		103/183	**1.41** **(1.12, 1.78)**	**1.42** **(1.12, 1.81)**	
GG	241/461				180/381				61/80			
Recessive	1,072/1,997			0.987	857/1,601			0.242	215/396			**0.015**
CC+CG	831/1,536	Reference	Reference		677/1,220	Reference	Reference		154/316	Reference	Reference	
GG	241/461	0.97(0.81, 1.15)	1.00(0.84, 1.20)		180/381	0.85(0.70, 1.04)	0.89 (0.72, 1.09)		61/80	**1.57** **(1.07, 2.30)**	**1.64 (1.10, 2.43)**	
Dominant	1,072/1,997			0.486	857/1,601			0.706	215/396			**0.019**
CC	280/538	Reference	Reference		229/405	Reference	Reference		51/133	Reference	Reference	
CG+GG	792/1,459	1.04(0.88, 1.23)	1.06(0.89, 1.27)		628/1,196	0.93(0.77, 1.12)	0.96 (0.79, 1.17)		164/263	**1.63** **(1.12, 2.37)**	**1.60 (1.08, 2.36)**	

*Adjusted for age, gender, smoking and drinking status.

† Adjusted for age, smoking and drinking status.

Significant results were highlighted in bold.

### *SERPINA6* mRNA levels in liver tissues from different genotype carriers

**Figure 2:**
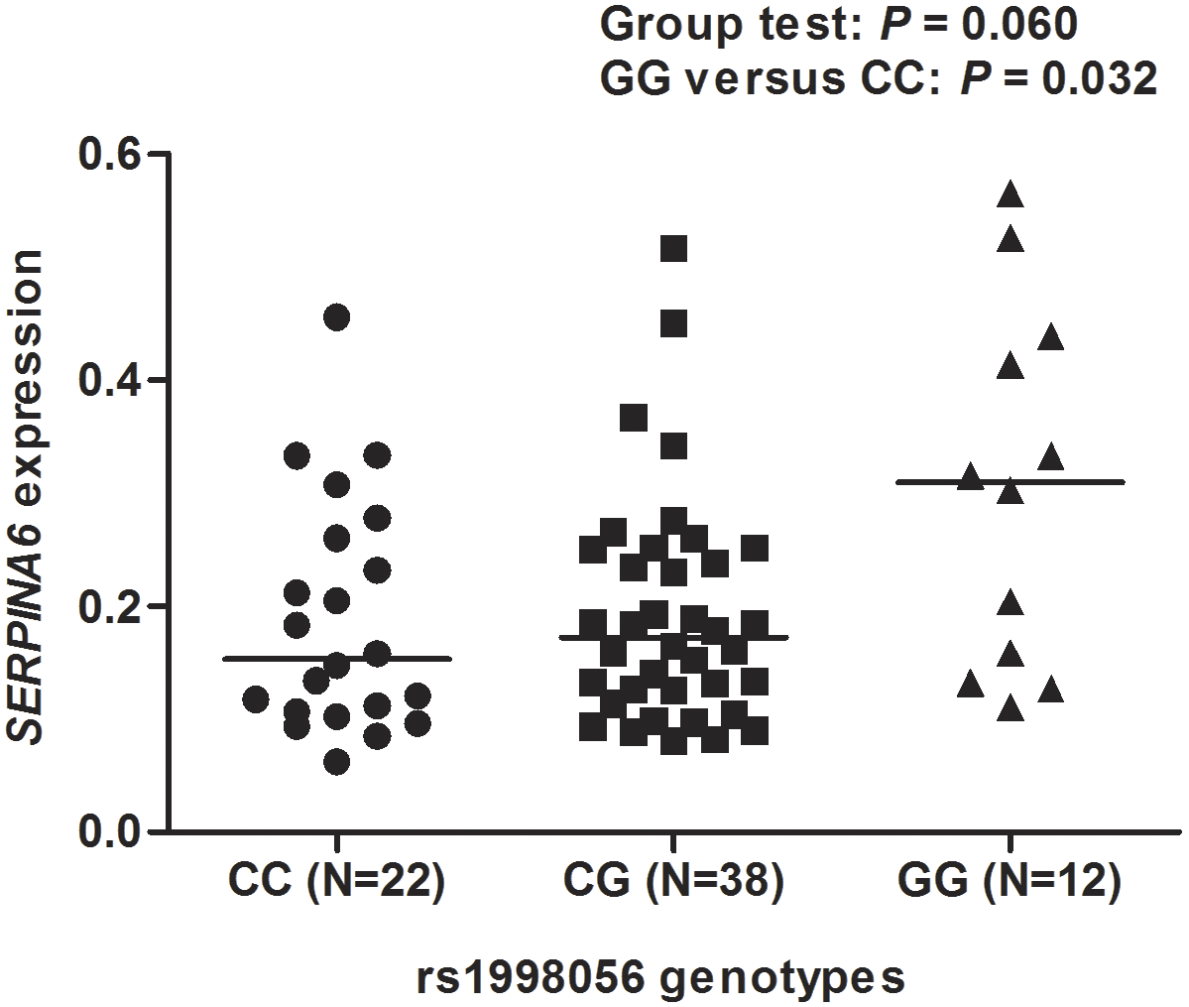
*SERPINA6* mRNA levels in liver tissues from different genotype carriers of rs1998056. Vertical scatter plots indicated relative gene expression of *SERPINA6* for 22 CC, 38 CG and 12 GG carriers. Horizontal line indicated median mRNA level of each genotype group.

To examine the effect of rs1998056 on *SERPINA6* expression in the target tissues, the levels of *SERPINA6* mRNA in normal liver tissues adjacent to the tumors from 72 individuals were quantitated by qPCR (Figure 2). We observed a borderline significance of *SERPINA6* expression among the CC, CG and GG genotype carriers (Kruskal-Wallis one-way ANOVA test: *P* = 0.060). Specially, the GG genotype carriers had significantly higher *SERPINA6* mRNA levels than the CC genotype carriers (Mann-Whitney test: *P* = 0.032). We also compared the *SERPINA6* mRNA levels between men and women, but did not find any difference (*P* = 0.937).

## Discussion

In this study, we used an integrative genomic strategy to demonstrated that 1) a simple but effective approach can be applied to identify new HCC-related genes and variants by integrating ChIP-seq data, genome mapping and dbSNP database; 2) *SERPINA6-*rs1998056 is significantly associated with female HCC risk suggested by the subsequent case-control study; and 3) results of qPCR using target tissues further supported the functional effect of this variant.

Nowadays, more and more studies highlight that genetic variants at TFBS have the potential to affect the protein (transcriptional factors)-DNA (cis-regulatory elements) interaction and then modify the expression of target genes, thus involving in the development of complex diseases including cancers [18], [24]–[27]. On the basis of previous studies and findings from Li Z et al. [18], we successfully applied a bioinformatics analysis to identify 15 genetic variants at FOXA1/2 binding elements. These candidate variants are near or within genes’ promoter regions and co-regulated by FOXA/ERα, thus probably playing a role in the development of HCC, especially in women. Our subsequent case-control study further found that *SERPINA6*-rs1998056 was associated with female HCC risk. This identified gene *SERPINA6* is known to be regulated by ERα [28]–[30], which suggested that our approach was reliable.

*SERPINA6*-rs1998056 is a C/G variation mapped to chr14: 94789495 (GRCh37), which is located in the first intron of *SERPINA6* gene. Besides evidences in our study, many authoritative databases online have also provided strong evidences to support the potentially functional role of this variant. F-SNP (http://compbio.cs.queensu.ca/F-SNP/) and RegulomeDB (http://regulome.stanford.edu/index) all show that *SERPINA6*-rs1998056 likely to affect the transcriptional factors binding and thus regulating the expression of their target genes. Specifically in the HepG2 cell line, this variant area can be bound by other important transcriptional factors including EP300, HNF4A and CEBPB; epigenetic marks of H3k4me1, H3K4me3 and H3K9ac that highlight active gene promoters are also found in the region; this variant area also overlaps with DNase I hypersensitive site (DHS), which is a specific chromatin structure often indicating cis-regulatory elements. Similar results are also obtained from HaploReg v2 (http://www.broadinstitute.org/mammals/haploreg/haploreg.php) and UCSC Genome Browser (http://genome.ucsc.edu/cgi-bin/hgGateway). All the evidences above strongly support that *SERPINA6-*rs1998056 is located at the active cis-regulatory element and probably modifies the transcriptional level of *SERPINA6* in liver. Moreover, LiverAtlas database (http://liveratlas.hupo.org.cn/) shows that *SERPINA6* is a HCC significant gene and Cancer Gene Expression Database (CGED) (http://lifesciencedb.jp/cged/) suggests that this gene significantly differently expresses in HCC comparing with non-tumor liver tissues, which suggest a potential association of *SERPINA6* with hepatocarcinogenesis.

*SERPINA6* encodes the major transport protein for glucorticoids and progestins in the blood, not only regulating endopeptidase and proteolysis, but also playing a role in hormone-related pathways [28]–[32]. Current researches have suggested a potential role of *SERPINA6* in liver diseases. Decreased circulating SERPINA6 concentrations were observed in patients with liver cirrhosis [33], [34]. Changed expression of SERPINA6 could provide information about liver impairment [35]. Some studies have indicated an association between SERPINA6 and type 2 diabetes too [36], [37]. All these diseases above are important risk factors during hepatocarcinogenesis. Based on previous studies and our results, we speculated that *SERPINA6*-rs1998056 was likely to influence the co-regulation of FOXA1/2 and ERα, modulate the expression of *SERPINA6*, then play a role in damage of hormone homeostasis and finally increase the risk of female HCC.

Several limitations of our study should be acknowledged. First, other candidate variants were not for population association examination due to several practical reasons including low MAFs or probe designed failure. Second, some other risk factors (family history, hepatitis virus infection, obesity, etc.) were not fully collected during investigation, thereby resulting in insufficient adjustment for ORs of HCC risk. Finally, data about hepatitis virus B (HBV) infection were missing in more than half of participants, so we could not analyze the HBV status in this study. Independent replication studies and biochemical assays are warranted to verify our results.

In conclusion, our study suggested that *SERPINA6*-rs1998056 regulated by FOXA/ERα might be associated with female HCC risk by using an integrative genomic strategy. Our findings not only presented an effective way to excavate predisposing variants and genes in complex diseases, but also advanced our understanding of the genetic etiology of HCC.

## Supporting Information

Table S1 Candidate variants identified by bioinformatic procedure.
(DOC)
