# Supplementary material for: Integrative Genomic Analysis Identifies That SERPINA6-rs1998056 Regulated by FOXA/ERα Is Associated with Female Hepatocellular Carcinoma
Source: PLoS One. 2014 Sep 8;9(9):e107246. doi: 10.1371/journal.pone.0107246 (PMC4157870; doi:10.1371/journal.pone.0107246)
Supplement: Table S1 — Candidate variants identified by bioinformatic procedure. (DOC) [file pone.0107246.s001.doc]

**Table S1.** Candidate variants identified by bioinformatic procedure.

| Loci^*^ | Chr: position | Ref. allele | GMAF^†^ | Gene |
| --- | --- | --- | --- | --- |
| rs4915432 | chr1 : 200342007 | T | NA | *LINC00862* |
| rs140034332 | chr3 : 193849667 | T | G=0.0005/1 | *HES1* |
| rs144000618 | chr3 : 193849674 | T | C=0.0014/2 | *HES1* |
| **rs111570813** | chr10 : 7742348 | C | T=0.0560/122 | *ITIH2* |
| rs188396810 | chr10 : 7742349 | G | A=0.0005/1 | *ITIH2* |
| rs150987975 | chr14 : 75988085 | A | C=0.0032/7 | *BATF* |
| rs34410720 | chr14 : 31927414 | -/C | NA | *DTD2* |
| rs190991647 | chr14 : 31927416 | A | G=0.0014/2 | *DTD2* |
| rs57571121 | chr14 : 94789449 | C | A=0.0257/56 | *SERPINA6* |
| rs142418468 | chr14 : 94789488 | A | G=0.0018/4 | *SERPINA6* |
| **rs1998056** | chr14 : 94789495 | C | C=0.4743/1032 | *SERPINA6* |
| rs10712625 | chr15 : 93425132 | TG | NA | *LOC100507217* |
| rs35335725 | chr17 : 39215489 | A | TG=0.1139/248 | *KRTAP2-3* |
| rs71300026 | chr17 : 39215493 | A | NA | *KRTAP2-3* |
| rs66461922 | chr17 : 39215490 | T | NA | *KRTAP2-3* |

^*^ Variants selected for genotyping were highlighted in bold. Specifically, only rs1998056 TaqMan® SNP Genotyping Assay was successfully designed and synthesized for the following validation.

^†^ GMAF was global minor allele frequency from 1000 Genomes data.

Abbreviations: NA, not available; *LINC00862*, long intergenic non-protein coding RNA 862; *HES1*, hairy and enhancer of split 1, (Drosophila); *ITIH2*, inter-alpha-trypsin inhibitor heavy chain 2; *BATF*, basic leucine zipper transcription factor, ATF-like; *DTD2*, D-tyrosyl-tRNA deacylase 2 (putative); *SERPINA6*, serpin peptidase inhibitor, clade A (alpha-1 antiproteinase, antitrypsin), member 6; *LOC100507217*, uncharacterized LOC100507217; *KRTAP2-3*, keratin associated protein 2-3.
